# Machine Learning Techniques for Identifying Lifestyle Factors Associated With Low Back Pain in Adults Aged 50 and Older Using Data From the Korean National Health and Nutrition Examination Survey

**DOI:** 10.1111/nhs.70248

**Published:** 2025-11-04

**Authors:** Songhee Ko, Heesung Yang, Namsu Kim, Kibong Choi, Hae‐Young Kim, Kyounghae Kim

**Affiliations:** ^1^ College of Nursing, Korea University Seoul South Korea; ^2^ Graduate School of Interdisciplinary Data Science Korea University Seoul South Korea; ^3^ Department of Public Health Sciences, Graduate School and Transdisciplinary Major in Learning Health Systems Seoul South Korea; ^4^ Department of Integrative Medicine, Major in Digital Healthcare Yonsei University College of Medicine Seoul South Korea; ^5^ Department of Health Policy and Management, College of Health Science Korea University Seoul South Korea

**Keywords:** chronic low back pain, lifestyle, machine learning

## Abstract

Chronic low back pain (cLBP) is shaped by multiple lifestyle factors, yet models with practical behavioral cutoffs remain scarce. This study developed a machine learning model to identify key lifestyle factors and specific thresholds linked to cLBP in adults aged 50 and older, using data from 5607 participants in the Korean National Health and Nutrition Examination Survey. Machine learning algorithms were trained and validated, with performance assessed via AUROC and SHAP for interpretability. The logistic regression model performed best (AUROC = 0.721, 95% CI: 0.699–0.742). SHAP analysis revealed that higher cLBP risk was associated with older age, female gender, prolonged sitting (≥ 6 h/day), low walking frequency (< 4–5 times/week), infrequent strength training (< 1 time/week), moderate‐intensity work, elevated stress, and smoking over five packs lifetime. Diet also mattered: cLBP risk rose among those dining out less than ~2.2 times/week, consuming under 2.9 servings/day of protein, or with carbohydrate intake outside 55%–65% of total energy. These practical cutoffs can help clinicians identify high‐risk individuals through simple assessments, guiding tailored interventions in physical activity, diet, smoking cessation, and stress management to prevent cLBP.

**Summary**

- Machine learning‐based analysis identified key modifiable lifestyle factors, such as physical inactivity, prolonged sitting, poor dietary habits, and smoking, that contribute to chronic low back pain (cLBP).
- Encouraging movement and balanced nutrition can mitigate cLBP risk, while prolonged sitting and moderate‐intensity work activities increase susceptibility.
- Healthcare providers should integrate lifestyle modifications into preventive strategies, emphasizing smoking cessation, regular physical activity, and dietary improvements to support individuals at risk of cLBP.

## 1 Introduction

In 2020, it was estimated that approximately 7.5% of the global population experienced lower back pain (LBP) (Stevans et al. 2021). Approximately 32%–42% of LBP cases progress from acute to chronic LBP (cLBP), which is defined as pain lasting for more than 3 months (Burke et al. 2024). Individuals with cLBP often fall into a vicious cycle in which they avoid movement and become depressed, leading to worsening pain, impaired function, and decreased quality of life (Slepian et al. 2019). These trajectories of cLBP result in heightened medical costs, equivalent to 1%–2% of the gross domestic product (Fatoye et al. 2023). An alternative to costly and modest management of these patients is secondary prevention, which involves identifying factors that aggregate LBP to become chronic and designing an appropriate management plan based on these factors to prevent progression to cLBP.

Accurate diagnosis and timely treatment are required to hinder frequent relapses and progression to cLBP. However, approximately 90% of cLBP cases are classified as non‐specific, meaning they lack a clear etiology, such as infection, tumor, or fracture (Maher et al. 2017). Current clinical guidelines for managing cLBP emphasize a comprehensive, multidisciplinary approach that integrates pharmacological and non‐pharmacological interventions (Foster et al. 2018). The U.S. Centers for Disease Control and Prevention (CDC) and the American College of Physicians (ACP) recommend prioritizing non‐pharmacological therapies before initiating pharmacological treatment (Dowell et al. 2022). These recommendations are supported by evidence indicating that psychosocial factors significantly delay recovery and reduce treatment efficiency in chronic pain conditions (Mescouto et al. 2022). Consequently, current guidelines endorse a combination of pharmacological options—such as acetaminophen, non‐steroidal anti‐inflammatory drugs (NSAIDs), tricyclic antidepressants, and muscle relaxants—with non‐pharmacological strategies including exercise, lifestyle modifications, psychological counseling, and weight management (Dowell et al. 2022; Knezevic et al. 2021; Orrillo et al. 2022).

Lifestyle, a modifiable factor, has been identified as a key component in diagnosing and treating chronic diseases, including cLBP. This reflects one of the health concepts from the 1974 Lalonde report and one of the five major health indicators identified in Healthy People 2030 (Pronk et al. 2021; Tulchinsky 2018). Previous research has established that various lifestyles are associated with cLBP, including smoking, sedentary behavior, and dietary habits, such as consuming a proinflammatory diet and reduced physical activity (Dai et al. 2021; Kastelic et al. 2018; Shin et al. 2022). Moreover, highly complex relationships between these habits might result in low predictive ability from traditional statistical methods, which seek simple linear algorithms (Franklin et al. 2017). Research on the relationship between lifestyle and cLBP is clouded in observational studies by inherent biases, including residual confounding, which complicates the interpretation of the results regarding the relationship between lifestyles and cLBP. To address these challenges, this study applied machine learning (ML) techniques with carefully selected factors identified from existing literature and chosen through a multicollinearity analysis. ML techniques are widely recognized as powerful tools for developing predictive models and analyzing data. These techniques can uncover hidden relationships and complex patterns within vast, high‐dimensional datasets, capturing complex non‐linear relationships and significantly improving prediction accuracy compared to traditional statistical methods (Wang and Alexander 2020).

A previous study utilized ML techniques to develop classification models, including logistic regression, k‐nearest neighbors, and Naive Bayes, based primarily on sociodemographic factors and comorbidities to predict the occurrence of cLBP (Shim et al. 2021), setting aside modifiable factors that are fundamental to the trajectory of cLBP. Moreover, ML models can effectively identify and suggest the importance levels of various modifiable lifestyle factors in predicting the development of cLBP.

This study aimed to develop a predictive model focusing on modifiable lifestyle factors. It sought to effectively identify the varying levels of importance of numerous lifestyle factors in predicting the onset of cLBP and suggest directions for modification, thereby laying the foundation for self‐management interventions for individuals at risk of cLBP.

## 2 Materials & Methods

### 2.1 Study Design

**FIGURE 1:**
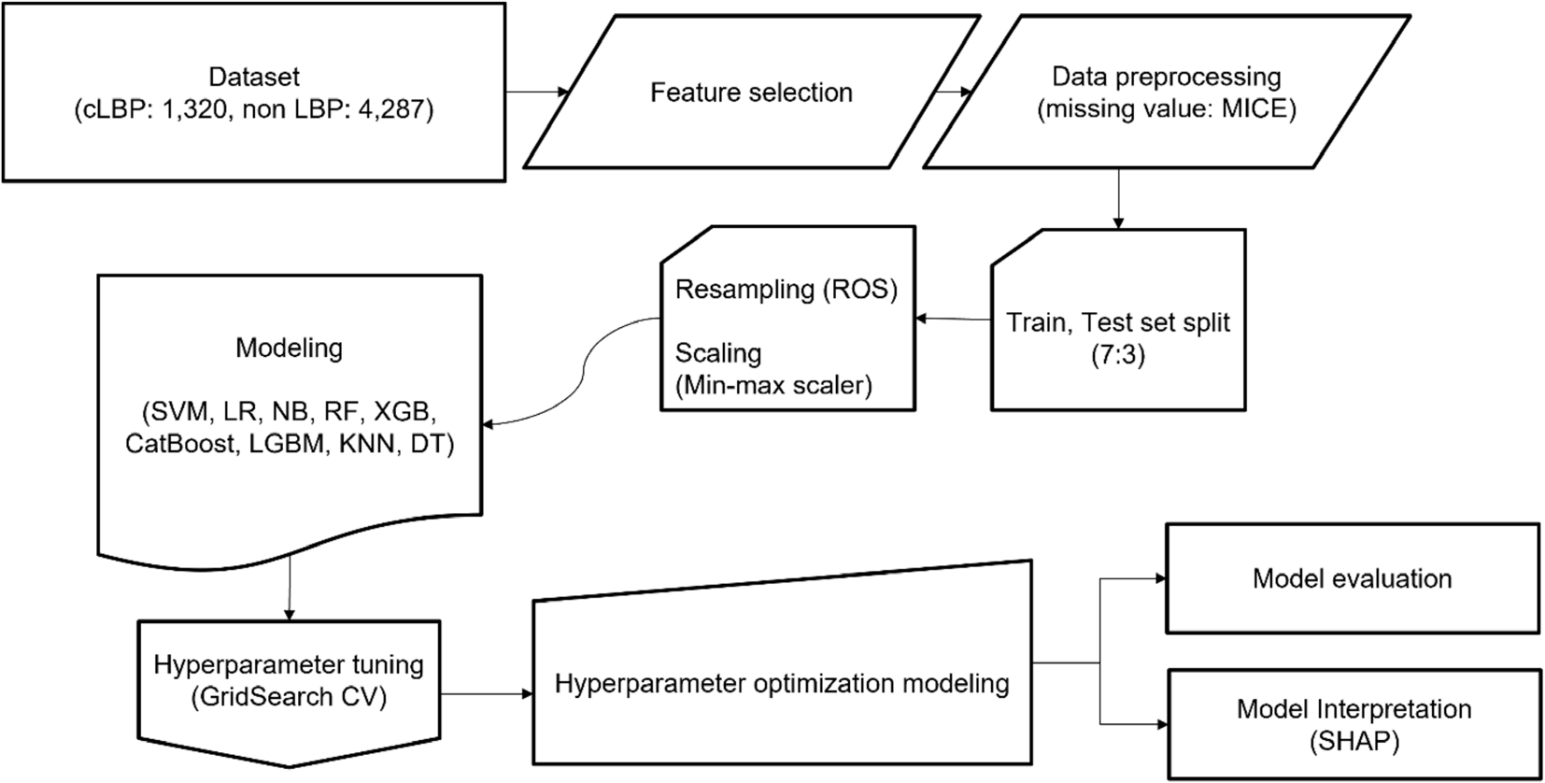
Flowchart of a machine learning process. ROS, random over sampler.

This study adopted a cross‐sectional, correlational study design to develop ML algorithm models to identify key determinants of lifestyle factors associated with cLBP. The overall workflow of the study, including data preprocessing, model building, and evaluation procedures, is illustrated in Figure 1.

### 2.2 Sample and Settings

The Korea National Health and Nutrition Examination Survey (KNHANES) is a nationwide cross‐sectional survey conducted annually by the Korea Centers for Disease Control and Prevention (KCDC) based on the National Health Promotion Act. KNHANES has assessed the health and nutritional status of nationally representative non‐institutionalized civilians in Korea since 1998 (Kweon et al. 2014).

**FIGURE 2:**
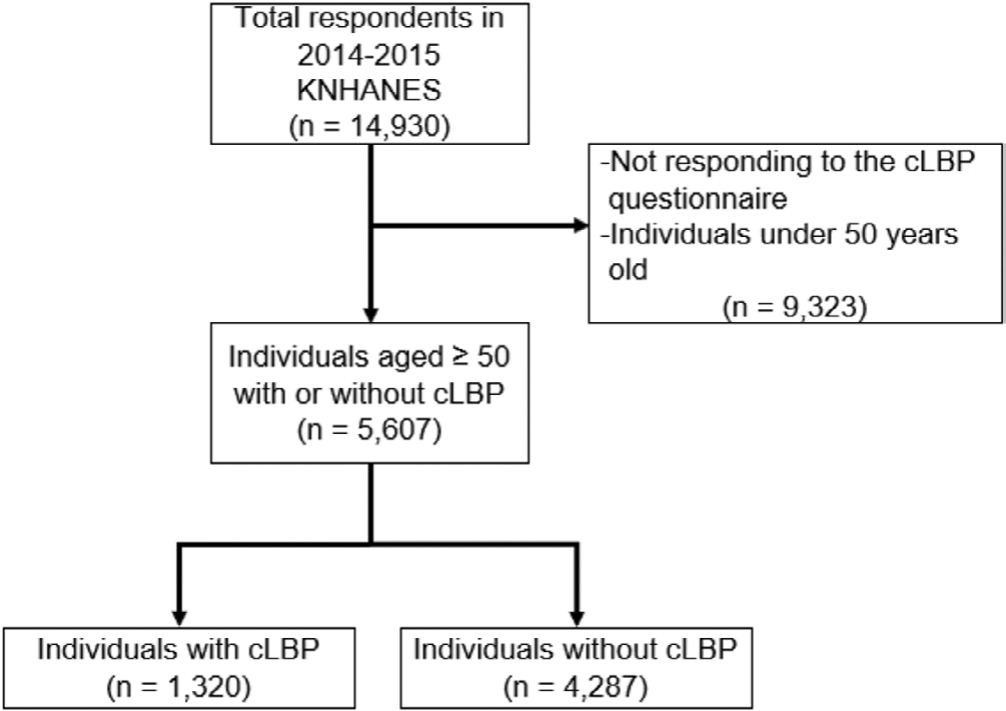
Data selection flow chart.

For this research, data collected during the sixth KNHANES, spanning 2014 to 2015, the latest KNHANES available for this study because it included data on the presence or absence of cLBP, were considered. Of 22 948 respondents targeted in the sixth KNHANES between 2014 and 2016, 14 930 participants who completed the survey in 2014 and 2015 were included in this study because the survey questions used to measure the lifestyle factors in this study were consistent in 2014–2015. However, changes were observed in the 2016 survey, ensuring variable consistency. According to the Global Burden of Disease study, LBP affects individuals across all age groups; however, its prevalence and incidence peak between ages 50 and 54 and continue to increase with age (Cheng et al. 2025). Therefore, including individuals aged ≥ 50 was appropriate for addressing the study objective. Consequently, 9323 individuals who did not respond to the cLBP inquiry or were < 50 years of age were excluded. As shown in Figure 2, the final dataset comprised 5607 respondents who satisfied all inclusion and exclusion criteria.

The KNHANES collects health, dietary, and lifestyle data using standardized national protocols. In the sixth KNHANES, dietary data were obtained through face‐to‐face interviews conducted by trained registered dietitians using the 24‐h dietary recall method and a semi‐quantitative food frequency questionnaire (FFQ). These dietitians followed standardized protocols and underwent rigorous pre‐survey training to ensure data accuracy and consistency (Korea Disease Control and Prevention Agency [KDCA] 2021).

### 2.3 Primary Outcomes and Lifestyle Factors

Based on the KNHANES 2014–2015 dataset, the variable selection process proceeded as follows: cLBP was employed as the primary outcome and defined as experiencing LBP for more than 30 days in the last 3 months.

This study selected the variables associated with LBP based on previous studies (Dai et al. 2021; Kastelic et al. 2018; Shin et al. 2022). Then, the variables were assessed for multicollinearity using correlation coefficients and variance inflating factors (VIFs) analysis, with VIF values of 10 or higher (Cheng et al. 2022). Of 34 variables initially selected, seven were removed due to multicollinearity issues such as meal, energy, and total vegetable intakes.

Physical activity variables were assessed through self‐reported responses using the Korean version of the Global Physical Activity Questionnaire (GPAQ), which demonstrated reliability, validity, and feasibility in population‐based studies in Korea (Jeon et al. 2013). This tool captures multiple domains of activity, including work, transport, and leisure, and provides standardized measures of physical activity levels.

Twenty‐seven lifestyle‐related variables were categorized into four groups based on the “health behaviors” domain within the five major health indicators—health status, health behaviors, healthcare access, social determinants of health, and public health infrastructure—as defined in *Healthy People 2030*, published by the World Health Organization and the U.S. Department of Health and Human Services (U.S. Department of Health and Human Services 2020; Centers for Disease Control and Prevention 2024). This framework facilitated comprehensive yet structured interpretation. The groups included: two sociodemographic variables (sex, age), 13 diet‐related lifestyle categories (dietary control, fat intake, meat intake, total vegetable intake, total fruit intake, mixed grain intake, milk intake, salt intake, carbohydrate intake, not skipping breakfast, dining out, water intake, and alcohol intake), which were evaluated by intake frequency and against recommended levels and total caloric intake using components of the Korean Healthy Eating Index (KHEI) (Yun and Oh 2019), nine physical activity‐related variables (walking, strength training, and flexibility exercises [each assessed weekly]; high‐ and moderate‐intensity activities during both leisure and work; sitting duration; and self‐reported activity limitations), and three additional variables (sleep duration, smoking, and stress level). However, this categorization was not applied during the analysis.

The variables were then refined to match the input format of the ML model. Binary responses were transformed to 0 for a negative response and 1 for a positive response. Numerical variables were redefined so that their quantitative values were consistent with their qualitative meaning. For example, in the frequency of alcohol consumption over a year, 0: not drinking, 1: less than once a month, 2: about once a month, 3: 2–4 times a month, 4: 2–3 times a week, 5: more than four times a week (Table S1).

### 2.4 Data Gathering and Preprocessing

Data processing was performed using SPSS IBM 27.0 Statistics software. The data collected from the sixth KNHANES underwent a rigorous preprocessing phase involving procedures such as managing missing data, feature selection, and transformation to ensure data quality and suitability for analysis. All missing values were imputed using Multivariate Imputation by Chained Equations (MICE), a widely used multiple imputation method that iteratively estimates each variable with missing data using the observed values of the other variables (Azur et al. 2011). Features about lifestyle factors and cLBP were chosen for inclusion in the study. To address the class imbalance issue, where 1320 people were categorized as experiencing cLBP and set to 1, while 4287 people had no cLBP and were set to 0, the training dataset underwent resampling techniques using a random over sampler (Han et al. 2005). To ensure consistent scaling, we employed the Min–Max scaler (Raju et al. 2020) to standardize the features to a common range and preserve the relationships between data points. Furthermore, the dataset was divided into training and testing subsets to support ML model development and evaluation.

### 2.5 Training and Test Dataset

The ML performance was assessed using the holdout technique, which involves an out‐of‐sample evaluation. This technique divided the dataset into two segments: 70% for training and 30% for testing. A grid search that used a 10‐fold cross‐validation method was also employed to establish the most effective ML model (Nasiriany et al. 2019).

### 2.6 ML Model Building and Evaluation

Various ML classifiers, including logistic regression, support vector machine, K‐nearest neighbors, Naive Bayes, decision trees, random forest, XGBoost, LightGBM, and CatBoost, were selected for model development. Detailed descriptions and model simulation parameters for each classifier are briefly explained in Table S2 (Nasiriany et al. 2019).

### 2.7 Performance Evaluation

The performance of each classification model was evaluated using the test dataset. The classifiers were compared based on the values of the area under the receiver operating characteristic curve (AUROC), accuracy, precision, and recall. Model performance was interpreted according to AUROC values: below 0.6 was considered poor, between 0.6 and 0.7 was moderate or fair, between 0.7 and 0.8 was good, between 0.8 and 0.9 was very good, and above 0.9 was excellent (de Hond et al. 2022).

### 2.8 Feature Importance and Interpretability

Permutation feature importance analysis was conducted to specify key factors influencing cLBP and the relative importance of each feature in the predictive models (Altmann et al. 2010). A thorough feature importance analysis was performed to select superior performance models, including logistic regression, SVM, and random forest.

An analysis of SHapley Additive exPlanations (SHAP) was conducted to explain important predictors. The SHAP value plot was used to specifically present the relationship between variables and the occurrence of cLBP. In the SHAP plot, the red color was interpreted as an increase (positive sign) or decrease (negative sign) in the occurrence of cLBP, with low values (or frequency) for each variable on the left and high values (or frequency) on the right, based on a reference point of 0 (Lundberg and Lee 2017). Additionally, to further illustrate how continuous predictors affect model outputs and to determine potential cutoff points, SHAP dependence scatterplots with overlaid density plots were generated for key continuous variables. These plots visualize both the distribution of the variable values and their corresponding SHAP values, thereby facilitating identification of inflection points where the predicted risk of cLBP notably shifts. A horizontal dashed line at SHAP = 0 represents no effect on model output, with values above or below the line indicating positive or negative contributions, respectively.

## 3 Results

### 3.1 Participant's Characteristics

Of the 5607 adults aged 50 and older who responded to the survey, 1320 patients (23.5%) experienced cLBP. The mean age of the participants was 64.32, with a standard deviation of 9. Slightly more than half were female (57.4%, *n* = 3221), had an education level of less than high school graduate (59.6%, *n* = 3305), and were employed (50.3%, *n* = 2787). Most were married (98.8%, *n* = 5542), dined out at least once a week (53.9%, *n* = 2763), did not attempt to control their diets (77.3%, *n* = 3958), had not performed strength exercises in the past week (77.4%, *n* = 4416), and had no activity limitations (88.8%, *n* = 4911). The average sedentary time and sleep were 7.2 (±3.6) h and 6.6 (±1.5) h, respectively. Differences in participants' demographic and lifestyle variables between the cLBP group and the non‐LBP group are summarized in Table 1. Briefly, individuals with cLBP were more likely to be female, had lower education levels, were unemployed, consumed fewer calories and more alcohol, and did not engage in medium‐intensity leisure time activities.

**TABLE 1:** Demographic data and variable features of the included population.

Variable		Total (*n* = 5607)	cLBP (*n* = 1320)	Non‐cLBP (*n* = 4287)	*p*
		*n* (%)	*n* (%)	*n* (%)	
Demographic characteristics					
Age, years (mean ± SD)		64.32 (9)	67.32 (9.14)	63.39 (8.71)	< 0.001
Sex	Male	2386 (42.6)	341 (14.3)	2045 (85.7)	< 0.001
	Female	3221 (57.4)	979 (30.4)	2242 (69.6)	
Diet‐related lifestyle					
Water intake, cup (mean ± SD)		5.34 (2.7)	4.37 (2.5)	4.63 (2.7)	0.003
Breakfast intake^a^ (mean ± SD)		9.01 (2.7)	9.04 (2.7)	9 (2.7)	0.662
Mixed grain intake^a^ (mean ± SD)		2.72 (2.2)	2.60 (2.2)	2.76 (2.2)	0.028
Total fruit intake^a^ (mean ± SD)		2.80 (2.2)	2.76 (2.2)	2.82 (2.2)	0.448
Total vegetable intake^a^ (mean ± SD)		3.77 (1.4)	3.64 (1.5)	3.81 (1.4)	< 0.001
Meat, fish, egg, and bean intake^a^ (mean ± SD)		6.52 (3.3)	6.06 (3.5)	6.70 (3.3)	< 0.001
Milk and dairy intake^a^ (mean ± SD)		2.77 (4.3)	2.41 (4)	2.88 (4.3)	< 0.001
Salt intake^a^ (mean ± SD)		6.77 (3.4)	7.45 (3.1)	6.55 (3.4)	< 0.001
Carbohydrate intake^a^ (mean ± SD)		1.82 (2)	1.37 (1.9)	1.97 (2.1)	< 0.001
Fat intake^a^ (mean ± SD)		2.66 (2.2)	2.19 (2.2)	2.81 (2.2)	< 0.001
Dining‐out frequency	More than twice a day	205 (3.6)	23 (11.2)	182 (88.8)	< 0.001
	Once a day	408 (7.3)	46 (11.3)	362 (88.7)	
	5–6 times a week	431 (7.7)	67 (15.5)	364 (84.5)	
	3–4 times a week	451 (8)	88 (19.5)	363 (80.5)	
	1–2 times a week	1268 (22.6)	292 (23)	976 (77)	
	1–3 times a month	1518 (27.1)	410 (27)	1108 (73)	
	Almost never (< 1/month)	846 (15.1)	302 (35.7)	544 (64.3)	
	Missing value	480 (8.6)	92 (19.2)	388 (80.8)	
Dietary control	Yes	1161 (20.7)	280 (24.1)	881 (75.9)	0.699
	No	3958 (70.6)	947 (28.4)	3011 (71.6)	
	Missing value	488 (8.7)	93 (19.1)	395 (80.9)	
Alcohol frequency intake	Past year, no drinking	2145 (38.3)	610 (23.7)	1535 (76.3)	< 0.001
	Less than once a month	925 (16.5)	228 (24.6)	697 (75.4)	
	About once a month	459 (8.2)	106 (23.1)	353 (76.9)	
	2–4 times a month	836 (14.9)	143 (17.1)	693 (82.9)	
	2–3 times a week	653 (11.6)	101 (15.5)	552 (84.5)	
	More than 4 times a week	456 (8.1)	81 (17.8)	375 (82.2)	
	Missing value	133 (2.4)	51 (38.3)	82 (61.7)	
Physical activity‐related lifestyle					
Work: High intensity	Yes	89 (1.6)	29 (32.6)	60 (77.4)	0.037
	No	5475 (97.6)	1269 (23.2)	4206 (76.8)	
	Missing value	43 (0.8)	22 (51.2)	21 (48.8)	
Work: Moderate intensity	Yes	437 (7.8)	140 (32)	297 (68)	< 0.001
	No	5126 (91.4)	1158 (2.3)	3968 (97.7)	
	Missing value	44 (0.8)	22 (50)	22 (50)	
Leisure: High intensity	Yes	392 (7)	44 (11.2)	348 (88.8)	< 0.001
	No	5176 (92.3)	1257 (24.3)	3919 (75.7)	
	Missing value	39 (0.7)	19 (48.7)	20 (51.3)	
Leisure: Moderate intensity	Yes	1154 (20.6)	173 (15)	981 (85.0)	< 0.001
	No	4412 (78.7)	1127 (25.5)	3285 (74.5)	
	Missing value	41 (0.7)	20 (48.8)	21 (51.2)	
Weekly walking	Never walks	1228 (21.9)	371 (30.2)	857 (69.8)	< 0.001
	1 day	311 (5.6)	82 (26.4)	229 (73.6)	
	2 days	467 (8.3)	127 (27.2)	340 (72.8)	
	3 days	666 (11.9)	144 (21.6)	522 (78.4)	
	4 days	394 (7)	96 (24.4)	298 (75.6)	
	5 days	566 (10.1)	104 (18.4)	462 (81.6)	
	6 days	254 (4.5)	55 (20.6)	199 (79.4)	
	7 days (everyday)	1656 (29.5)	313 (18.9)	1343 (81.1)	
	Missing value	65 (1.2)	28 (43.1)	37 (56.9)	
Weekly strength exercise	Never	4416 (77.4)	1132 (25.6)	3284 (74.4)	< 0.001
	1 day	126 (2.6)	18 (14.3)	108 (85.7)	
	2 days	186 (3.7)	30 (16.1)	156 (83.9)	
	3 days	196 (3.8)	34 (17.3)	162 (82.7)	
	4 days	114 (2.2)	13 (11.4)	101 (88.6)	
	≥ 5 days	520 (10.3)	71 (13.7)	449 (86.3)	
	Missing value	49 (0.9)	22 (44.9)	27 (55.1)	
Weekly flexibility exercises	Never	2684 (47.9)	688 (25.6)	1996 (74.4)	0.002
	1 day	207 (3.7)	49 (23.7)	158 (76.3)	
	2 days	401 (7.2)	86 (21.4)	315 (78.6)	
	3 days	492 (8.8)	116 (23.6)	376 (76.4)	
	4 days	227 (4)	47 (20.7)	180 (79.3)	
	≥ 5 days	1544 (27.5)	311 (20.1)	1233 (79.9)	
	Missing value	52 (0.9)	23 (44.2)	29 (55.8)	
Activity limitation	Yes	685 (12.2)	340 (49.6)	345 (50.4)	< 0.001
	No	4911 (87.6)	973 (19.8)	3938 (80.2)	
	Missing value	11 (0.2)	7 (63.6)	4 (36.4)	
Sitting time, hours (mean ± SD)		7.2 (3.6)	7.8 (3.8)	7 (3.5)	< 0.001
Other lifestyles					
Smoking	Never smoked, < 100 cigarettes	3397 (60.6)	917 (27)	2480 (73)	< 0.001
	≥ 100 cigarettes	2064 (36.8)	343 (16.6)	1721 (83.4)	
	Missing value	146 (2.6)	60 (41.1)	86 (58.9)	
Perceived stress level	Rarely	1376 (24.5)	252 (18.3)	1124 (81.7)	< 0.001
	Sometimes	2992 (53.4)	638 (21.3)	2354 (79.7)	
	Often	860 (15.3)	280 (32.6)	580 (67.4)	
	Very often	230 (4.1)	89 (38.7)	141 (61.3)	
	Missing value	149 (2.7)	61 (40.9)	88 (59.1)	
Average sleep time per day, hours (mean ± SD)		6.6 (1.5)	6.4 (1.7)	6.7 (1.4)	< 0.001

^a^ Variable assessed using the Korean Healthy Eating Index for Adults.

### 3.2 Model Performance

**FIGURE 3:**
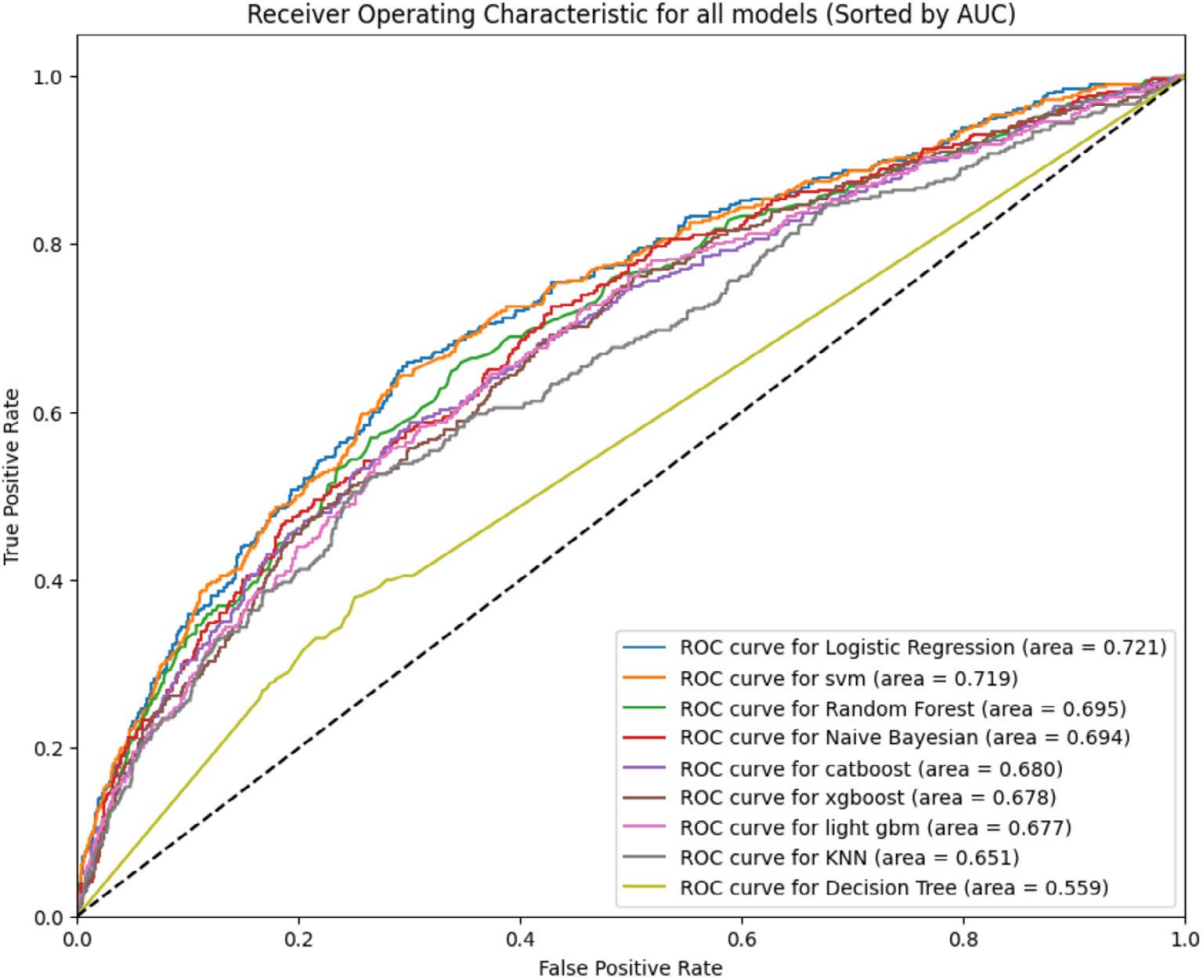
AUROC for all test data. CatBoost, category boosting; KNN, K‐nearest neighbor; LightGBM, light gradient‐boosting machine; SVM, support vector machine; XGBoost, extreme gradient boosting.

After applying the test dataset to all ML techniques to predict cLBP occurrence, the performance of the ML models is detailed in Table 2. The logistic regression machine model demonstrated the best performance among the nine ML algorithms for predicting cLBP. The support vector machine model showed a slightly lower AUROC than the logistic regression model (AUROC = 0.719, 95% CI = 0.698–0.741), with the decision tree model exhibiting the worst performance (AUROC = 0.563, 95% CI = 0.539–0.587). Additionally, the accuracy, precision, and recall metrics of each model were evaluated. According to Table 2, the logistic regression model has the highest AUROC, and the remaining performance is suboptimal (accuracy = 0.683, 95% CI = 0.661–0.706; precision = 0.392, 95% CI = 0.369–0.415). The random forest model, which ranked third in AUROC performance, showed superior results in accuracy (0.772, 95% CI = 0.752–0.792) and precision (0.514, 95% CI = 0.49–0.537). The logistic regression model achieved the highest recall performance (0.664, 95% CI = 0.642–0.687). Based on these performances, the logistic regression model was deemed most suitable for subsequent variable analyses, as illustrated by the ROC curves in Figure 3.

**TABLE 2:** Performance of all machine learning models.

Model	AUROC	Accuracy	Precision	Recall
	(95% CI)	(95% CI)	(95% CI)	(95% CI)
Logistic regression	0.721 (0.699, 0.742)	0.683 (0.661, 0.706)	0.392 (0.369, 0.415)	0.664 (0.642, 0.687)
SVM	0.719 (0.698, 0.741)	0.689 (0.667, 0.711)	0.395 (0.372, 0.419)	0.644 (0.621, 0.666)
Random forest	0.695 (0.673, 0.717)	0.772 (0.752, 0.792)	0.514 (0.49, 0.537)	0.292 (0.271, 0.314)
Naive Bayesian	0.694 (0.672, 0.716)	0.634 (0.611, 0.657)	0.341 (0.318, 0.363)	0.621 (0.597, 0.644)
CatBoost	0.680 (0.658, 0.703)	0.753 (0.732, 0.773)	0.46 (0.431, 0.478)	0.333 (0.311, 0.356)
XGBoost	0.678 (0.656, 0.7)	0.738 (0.717, 0.759)	0.421 (0.397, 0.444)	0.346 (0.323, 0.369)
LightGBM	0.677 (0.654, 0.699)	0.706 (0.684, 0.728)	0.385 (0.361, 0.408)	0.449 (0.425, 0.472)
K‐nearest neighbor (KNN)	0.651 (0.628, 0.673)	0.635 (0.612, 0.658)	0.336 (0.314, 0.359)	0.592 (0.569, 0.616)
Decision tree	0.563 (0.539, 0.587)	0.655 (0.632, 0.677)	0.308 (0.286, 0.33)	0.392 (0.369, 0.416)

Abbreviations: AUROC, area under the receiver operating characteristic curve; CatBoost, category boosting; KNN, K‐nearest neighbor; LightGBM, light gradient‐boosting machine; SVM, support vector machine; XGBoost, extreme gradient boosting.

### 3.3 Rick Factor Analysis

**FIGURE 4:**
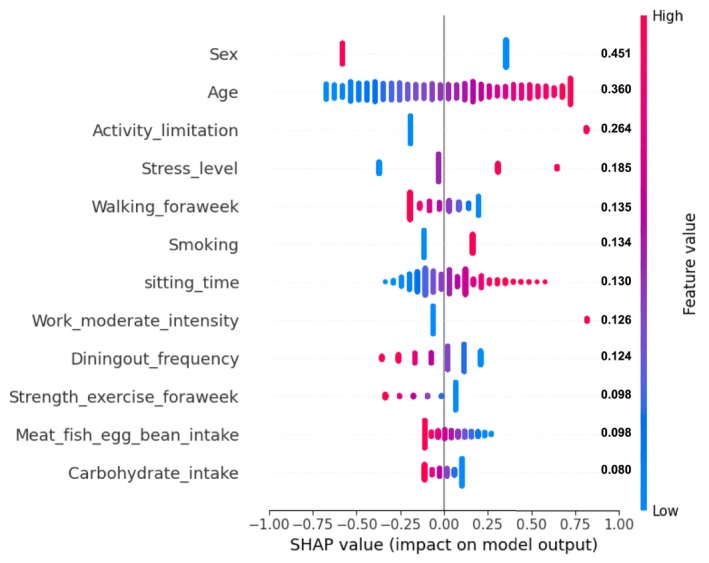
SHAP summary plot (bee swarm) for logistic regression. LR, logistic regression; SHAP, SHapley additive exPlanations.

The performance of the logistic regression model in predicting cLBP was quantified using the AUROC, after which the top 10 influential factors were elucidated using the SHAP values derived from the test set. A descending order was established for each variable based on the average impact on the model, as shown in Figure 4. The variable “sex” emerged as the most significant determinant, with a SHAP value of 0.451, indicating females are at higher risk for cLBP. It was followed by “age” and “activity limitation,” with values of 0.360 and 0.264, respectively, suggesting increased risk associated with limitation in physical activity and advancing age within the 50–80 years range.

“Stress level” (0.185) and low weekly walking frequency (0.135) exhibited relatively high SHAP values, indicating that both elevated psychological stress and reduced physical activity are key contributors to an increased risk of cLBP. Moreover, “smoking” (0.134) was positively associated with cLBP, with individuals who had smoked more than five packs (100 cigarettes) in their lifetime facing a higher risk than those who had never smoked or smoked less. Prolonged “sitting time” (0.142) and participation in “moderate‐intensity work” (0.118) were associated with an increased cLBP risk. Notably, a higher “dining out frequency” (0.124) was associated with greater risk, potentially reflecting suboptimal dietary patterns or weight gain. Lower levels of “weekly strength exercise” (0.098) and reduced intake of “meat, fish, eggs, and beans” (0.098) were associated with cLBP, suggesting that inadequate muscle‐strengthening activity and insufficient protein and nutrient intake may contribute to musculoskeletal vulnerability. Furthermore, “carbohydrate intake” (0.08) demonstrated that consuming less than the recommended amount was associated with a higher risk of cLBP, likely due to energy imbalance and muscle fatigue. In addition to these top‐ranking factors, the comprehensive SHAP summary plot illustrating all evaluated variables is presented in Figure S2. Permutation feature importance analyses further confirmed the influence of demographic, physical, and dietary variables across different ML models, as presented in Figure S1.

**FIGURE 5:**
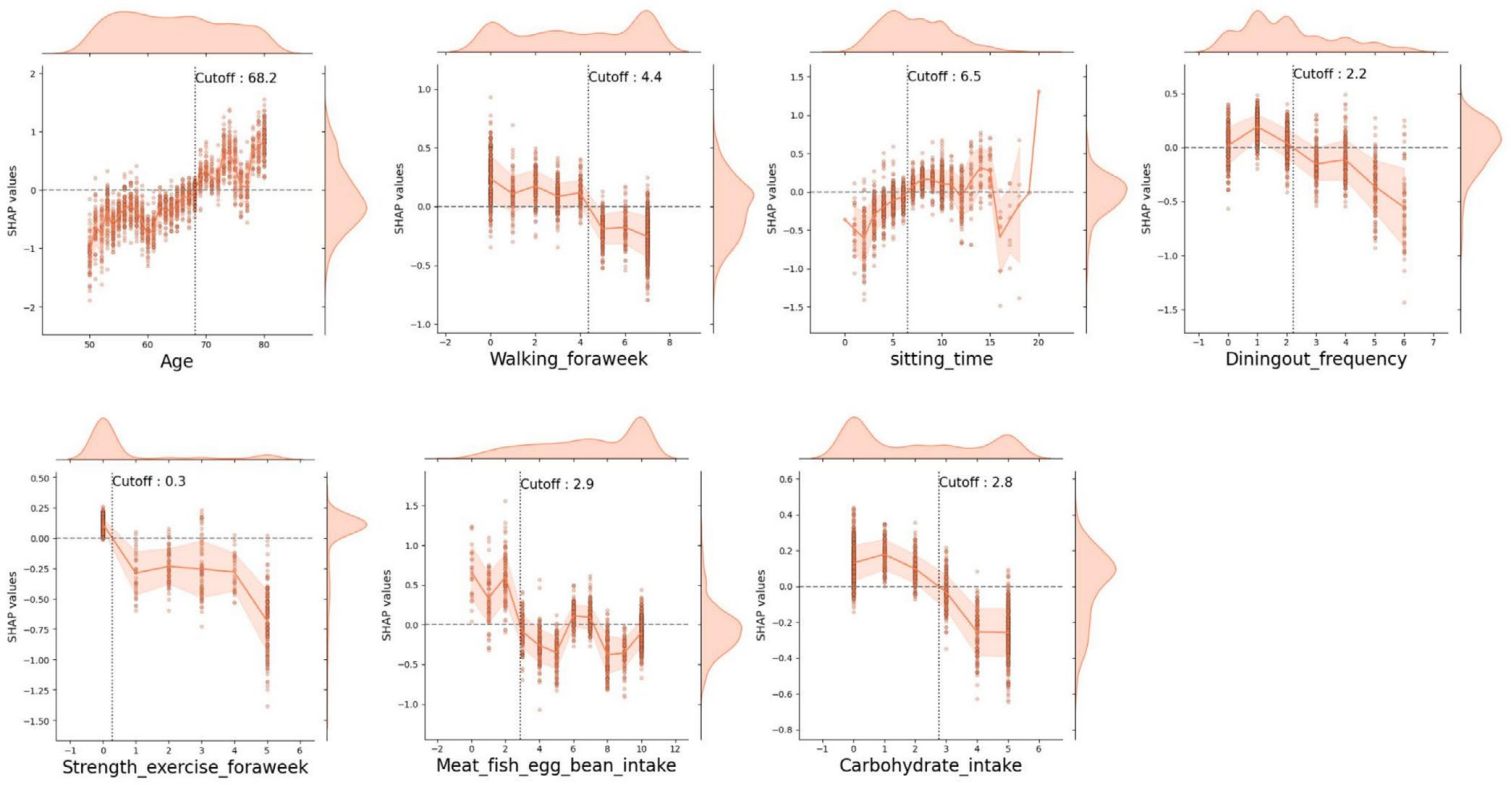
Seven examples of scatterplots showing the relationship between continuous predictors and model outputs (represented using SHAP values). Horizontal dash lines indicate no effect on model output, whereas above or below the line represents positive or negative effects, respectively. Density plots on the upper and right panels of each graph demonstrate the distributions of each predictor and the corresponding SHAP values.

To explore these associations in greater detail and identify actionable thresholds, SHAP dependence plots were constructed for continuous predictors such as age, sitting time, walking frequency, dining out frequency, strength exercise, meat/fish/eggs/beans intake, and carbohydrate intake. As shown in Figure 5, the scatterplots illustrate how SHAP values vary across the range of each variable, with dotted lines indicating estimated cutoff points (such as age: 68.2 years, walking frequency: 4.4 times/week, sitting time: 6.5 h/day, dining out: 2.2 times/week, protein intake: 2.9 servings/day, carbohydrate intake: 2.8 servings/day). These thresholds suggest levels at which cLBP risk increases significantly, offering practical targets for lifestyle interventions.

## 4 Discussion

We employed ML techniques to identify predictive lifestyle factors of cLBP in a representative sample of non‐institutionalized adults aged 50 and older. This study highlights a unique approach to variable selection in ML models, using VIF values and correlation analysis. Despite the benefits of feature selection in enhancing learning performance, computational efficiency, memory storage, and model generalization, it is often overlooked in ML research. This study proposed a novel experimental feature selection method to address multicollinearity (Lindner et al. 2022). Based on this approach, we captured complex non‐linear relationships, developed an ML model with strong predictive ability, and achieved an AUC of up to 0.721. Recognizing the difficulty in interpreting individual variables using only prediction metrics (Shim et al. 2021), we applied permutation feature importance and SHAP values to improve model interpretability. The final ML model and SHAP analysis underscored the significance of various lifestyle factors previously linked to cLBP and clarified how their implementation affects cLBP occurrence.

Sex and age played as the most critical predictors. LBP is more prevalent in women, likely due to occupational, hormonal, and neurophysiological factors (Bento et al. 2020), consistent with our finding that women had higher cLBP rates. Wong et al. (2017) noted that aging contributes via spinal degeneration, reduced activity, and cognitive changes, matching our results linking age with increased cLBP risk.

Beyond these demographic factors, psychosocial stress was a strong predictor in our model. Elevated stress levels activate the HPA axis, leading to chronic inflammation and heightened pain sensitivity (Borsook et al. 2012). Scott et al. (2017) found that stress and depression were closely tied to persistent cLBP, aligning with our findings that higher stress substantially increased cLBP risk.

Physical activity‐related factors were also identified as key predictors, reinforcing the link between insufficient movement and cLBP. For example, Kastelic et al. (2018) showed that prolonged sedentary behavior leads to poor posture, muscle weakness, and disc degeneration, raising LBP risk. This aligns with our finding that sitting over 6.5 h daily substantially elevated cLBP risk. Similarly, Mohan et al. (2018) observed reduced abdominal and respiratory muscle strength, along with decreased diaphragmatic mobility, in patients with cLBP, suggesting that such physiological changes weaken the core muscle group and may increase the risk of functional impairments associated with spinal stability. Accordingly, Sihinkiewicz et al. (2022) demonstrated strength training enhances core stability, reducing lumbar instability and chronic LBP, consistent with our observation that less than once‐weekly strength training increased risk. Tian and Meng (2019) also showed that regular stretching improves circulation and reduces stiffness, helping with pain relief. Our study similarly found that lower walking and strength exercise frequencies were linked to higher cLBP risk, underscoring how inactivity exacerbates spinal issues. This is consistent with previous findings suggesting that reduced physical activity in older adults contributes not only to functional decline but also to increased vulnerability to chronic health conditions, highlighting the importance of maintaining regular movement across various domains of life (Choi et al. 2013).

Our findings provided greater specificity by identifying approximately 4.4 walking sessions weekly as a threshold, beyond which cLBP risk increased. This moves beyond general advice, offering clinicians quantifiable targets. Interestingly, moderate‐intensity occupational activities, like brisk walking at work or caring for children > 10 min, showed even higher SHAP importance than exercise frequency, suggesting that repetitive daily tasks may not protect spinal health but could contribute to cLBP. This supports Heuch et al. (2017), who found heavy‐lifting occupations raised cLBP risk by 1.36 times in men and 1.30 times in women.

Smoking was another significant predictor. It impairs blood supply and accelerates disc degeneration, as Xu et al. (2023) noted. Dai et al. (2021) reported non‐smokers had a 7.8% lower back pain risk. Our results showed fewer than five lifetime packs or non‐smoking status substantially reduced cLBP risk, underscoring smoking avoidance's importance.

Dietary behaviors, though relatively lower in importance compared to other predictors, still demonstrated meaningful associations with cLBP. Interestingly, our study found that participants dining out fewer than approximately 2.2 times per week faced a higher risk of cLBP, a finding that contrasts with studies showing frequent dining out increases saturated fat and sodium intake, fostering proinflammatory diets and chronic disease risk (Abu Bakar et al. 2022; Ju 2020; Wu et al. 2023). This discrepancy may stem from the likelihood that individuals who dine out less frequently have lower socioeconomic status, reduced social activity, or avoid dining out due to existing health conditions. Supporting this, Matsuda et al. (2024) reported that older Japanese adults with chronic LBP and low daily step counts had a 1.89‐fold higher risk of social frailty, suggesting that diminished social participation and mobility associated with chronic pain could partly explain our findings.

We also found that consuming < 2.9 servings of protein daily significantly raised cLBP risk. This aligns with Korean dietary guidelines recommending ≥ 4 servings/day for men and ≥ 2.5 for women ≥ 65. Noh et al. (2022) reported Korean women over 50 consuming < 0.8 g/kg/day protein had a 1.83‐fold increased cLBP risk, rising to 2.91‐fold without exercise, emphasizing protein's role in preventing sarcopenia, improving lumbar stability, and reducing inflammation.

Carbohydrate intake relative to total energy also mattered. Participants below recommended 55%–65% energy from carbs had a greater cLBP risk. While most studies focus on excess carbs' proinflammatory effects, Boyacı et al. (2021) showed acute glucose swings trigger more oxidative stress than sustained hyperglycemia, suggesting inadequate carb intake could impair glycogen stores, cause rapid muscle fatigue, and raise LBP risk.

Using SHAP within ML, we identified how lifestyle and demographic factors—physical activity, diet, smoking, stress, age, sex—affect cLBP, establishing actionable thresholds: sitting < 6 h/day, walking ≥ 5 times weekly, strength training≥once weekly, dining out ≥ 2.2 times weekly, consuming ≥ 2.9 protein servings daily, carbs at 55%–65% energy, smoking < 5 lifetime packs, and lower stress. These offer valuable clinical guidance for identifying high‐risk individuals via simple lifestyle checks. They are particularly relevant for older adults, prone to lower food intake, physical activity, and social participation. These thresholds can inform screening and tailored interventions like diet counseling, exercise promotion, smoking cessation, and stress management. Unlike lab studies, our work analyzed non‐clinical lifestyle data via ML and presented directional impacts using SHAP, enhancing interpretability. As the model was developed in older adults, caution is needed before generalizing to younger groups. These findings nevertheless provide meaningful insights for clinical practice, offering a practical basis to identify high‐risk individuals and design personalized lifestyle interventions to prevent cLBP.

## 5 Limitations

This study has several constraints. First, it utilized a cross‐sectional survey of adults aged ≥ 50; therefore, the findings may not be generalized to younger individuals, those residing in institutional settings, or populations in different sociocultural or healthcare contexts. Second, the data drawn from the 2014–2015 cycle of the sixth KNHANES may not fully capture recent lifestyle shifts or public health policies, particularly those influenced by the coronavirus disease 2019 (COVID‐19) pandemic. However, key lifestyle variables—such as physical activity and sedentary behavior—have shown relative stability across KNHANES cycles before and after the pandemic, supporting the ongoing relevance of the model's predictors. Third, causality cannot be inferred from SHAP plots. In this study, while interpreting SHAP outputs, previous studies were referenced to substantiate the associations between lifestyle factors and cLBP onset. As with prior research relying on existing datasets, we were unable to include all possible predictors, including sleep quality, in developing the ML model to explain cLBP. Future research should incorporate more recent datasets and adopt longitudinal designs to confirm the stability of these associations and identify emerging risk factors post‐pandemic. This approach would also enhance causal inference and improve the generalizability of ML‐based predictive models.

## 6 Conclusions

This study represents a significant advancement in the secondary prevention of cLBP by demonstrating that personalized, non‐pharmacological interventions based on quantifiable lifestyle thresholds offer a practical and pragmatic alternative to traditional management strategies. By identifying specific thresholds for risk factors—including sitting more than 6 h daily, walking fewer than 4–5 times per week, engaging in strength training less than once weekly, participating in moderate‐intensity occupational tasks, consuming under 2.9 servings of protein daily, maintaining carbohydrate intake below 55%–65% of total energy, dining out fewer than approximately 2.2 times weekly, smoking more than five lifetime packs, experiencing high stress, along with older age and female sex—using logistic regression with SHAP analysis, this study emphasizes the value of tailoring preventive efforts to individual risk profiles. Such personalized approaches enable clinicians to target modifiable behaviors and mitigate the risk of cLBP more effectively. Using a large, nationally representative dataset strengthens the external validity of these findings, supporting their generalizability. However, as this study was cross‐sectional, longitudinal research is warranted to establish causality more definitively. These results highlight the potential of precise, lifestyle‐based interventions to reduce reliance on pharmacotherapy and improve long‐term musculoskeletal outcomes through early, community‐level implementation.

## 7 Relevance for Clinical Practice

The findings offer substantial implications for clinical practice, particularly for early identification and prevention of cLBP among middle‐aged and older adults. Unlike prior research that primarily offered broad, qualitative guidance, this study presents specific, actionable thresholds—such as sitting over 6 h daily, walking fewer than 4–5 times weekly, performing strength training less than once weekly, consuming fewer than 2.9 servings of protein daily, carbohydrate intake below 55%–65% of total energy, dining out less than 2.2 times weekly, smoking more than five lifetime packs, and elevated stress levels—that can inform personalized lifestyle counseling and risk stratification.

These cutoffs enhance understanding of how modifiable factors influence cLBP risk and offer practical benchmarks clinicians can use in routine assessments. During outpatient visits or health screenings, physicians and nurses could incorporate these variables into simple risk checklists or brief lifestyle questionnaires to efficiently identify high‐risk individuals. Patients reporting limited activity, inadequate diet, or elevated stress could then be prioritized for targeted interventions.

Building on this framework, clinicians might deliver tailored exercise prescriptions emphasizing walking and strength training, provide nutritional counseling to ensure adequate protein and macronutrient balance, and support smoking cessation and stress management. Integrating these thresholds into EHR systems as automated alerts could prompt providers to address lifestyle risks without added assessment burdens.

Beyond individual care, these findings highlight opportunities for community‐based approaches. Community health nurses could apply these criteria in local screenings to identify high‐risk adults and implement targeted programs—like group exercise, dietary workshops, and psychoeducation—to enhance self‐care capacity among community residents. In particular, because middle‐aged and older adults often experience declines in activity, nutrition, and social participation—factors linked to cLBP and frailty—community‐based strategies that support self‐care and promote healthy lifestyles may help alleviate this burden. Previous studies have demonstrated that engaging in self‐care behaviors and maintaining healthy lifestyle practices can play an important role in preventing physical decline and frailty among individuals with chronic conditions (Oh et al. 2024; Seomun et al. 2006; Tanimura et al. 2017). This suggests that the specific lifestyle thresholds proposed in this study may also be applicable to community‐based preventive nursing.

These quantifiable cutoffs from the ML model enhance preventive precision, enabling healthcare professionals to move beyond broad advice toward individualized, evidence‐based strategies. Incorporating personalized lifestyle assessments into routine practice may reduce cLBP prevalence, alleviate functional limitations, and improve healthcare resource efficiency.

## Author Contributions

**Songhee Ko:** conceptualization, methodology, writing – original draft. **Heesung Yang:** conceptualization, formal analysis, writing – original draft. **Namsu Kim:** conceptualization, writing – original draft; **Kibong Choi:** formal analysis, writing – review and editing. **Hae‐Young Kim:** conceptualization, writing – original draft, and writing – review and editing, supervision. **Kyounghae Kim:** conceptualization, writing – original draft, and writing – review and editing, supervision.

## Ethics Statement

This secondary analysis study used data from the 2014–2015 KNHANES and was exempt from review by the Institutional Review Board (IRB approval no: KUIRB‐2024‐0292‐01).

## Conflicts of Interest

The authors declare no conflicts of interest.

## Supporting information


**Table S1:** Refined value forms for machine learning.
**Table S2:** Description of the machine learning model.
**Figure S1:** The permutation importance feature.
**Figure S1:1** Logistic regression.
**Figure S1:2** Support vector machine (SVM).
**Figure S1:3** Random forest.
**Figure S1:4** Naive Bayesian.
**Figure S1:5** CatBoost.
**Figure S1:6** XGBoost.
**Figure S1:7** LightGBM.
**Figure S1:8** K‐nearest neighbor (KNN).
**Figure S1:9** Decision tree.
**Figure S2:** SHAP value plot of logistic regression.

**Data S1:** STROBE statement—Checklist of items that should be included in reports of cross‐sectional studies.

## Data Availability

The data underlying this article are available in the Korea National Health and Nutrition Examination Survey (KNHANES) repository, managed by the Korea Disease Control and Prevention Agency (KDCA), at https://knhanes.kdca.go.kr. The datasets were derived from sources in the public domain, specifically the 2014 Health Examination Survey, 2014 Health Interview Survey, 2014 Nutrition Survey, 2015 Health Examination Survey, 2015 Health Interview Survey, and 2015 Nutrition Survey, available at https://knhanes.kdca.go.kr/knhanes/rawDataDwnld/rawDataDwnld.do.
