# Supplementary material for: Machine Learning Techniques for Identifying Lifestyle Factors Associated With Low Back Pain in Adults Aged 50 and Older Using Data From the Korean National Health and Nutrition Examination Survey
Source: Nurs Health Sci. 2025 Nov 4;27(4):e70248. doi: 10.1111/nhs.70248 (PMC12584052; doi:10.1111/nhs.70248)

| **Categories** | **Variables** | **Refined value forms for ML and ranges** | **Type** |
| --- | --- | --- | --- |
| *Demographic characteristics* | Sex | 0: male, 1: female | Categorical |
|  | Age, years | Range: 50–80 | Numerical |
| *Diet-related lifestyle* | Water intake | Range: 0–30 | Numerical |
|  | Breakfast frequency | Range: 0–10 | Numerical |
|  | Mixed grain intake | Range: 0–5 | Numerical |
|  | Total fruit intake | Range: 0–5 | Numerical |
|  | Total vegetable intake | Range: 0–5 | Numerical |
|  | Meat, fish, egg, bean intake | Range: 0–10 | Numerical |
|  | Milk and dairy intake | Range: 0–10 | Numerical |
|  | Salt intake | Range: 0–10 | Numerical |
|  | Carbohydrate intake | Range: 0–5 | Numerical |
|  | Fat intake | Range: 0–5 | Numerical |
|  | Dining-out frequency | 0: almost never (less than once a month)  1: 1-3 times a month  2: 1-2 times a week  3: 3-4 times a week  4: 5-6 times a week  5: once a day  6: more than twice a day | Categorical |
|  | Dietary control | 1: yes, 2: no | Categorical |
|  | Alcohol intake frequency | 0: did not drink within the past year  1: less than once a month  2: about once a month  3: 2-4 times a month  4: 2-3 times a week  5: more than 4 times a week | Categorical |
| *Physical activity-related lifestyle* | Work: high intensity | 0: not engaged, 1: engaged | Categorical |
|  | Work: moderate intensity | 0: not engaged, 1: engaged | Categorical |
|  | Leisure: high intensity | 0: not engaged, 1: engaged | Categorical |
|  | Leisure: moderate intensity | 0: not engaged, 1: engaged | Categorical |
|  | Weekly walking | 0: never  1: one day a week  2: two days a week  3: three days a week  4: four days a week  5: five days a week  6: six days a week  7: all week | Categorical |
|  | Weekly strength exercise | 0: never  1: one day a week  2: two days a week  3: three days a week  4: four days a week  5: more than five days a week | Categorical |
|  | Weekly flexibility exercises | 0: never  1: one day a week  2: two days a week  3: three days a week  4: four days a week  5: more than five days a week | Categorical |
|  | Activity limitation | 0: no limitation, 1: limitation present | Categorical |
|  | Sitting time | Range: 0–20 | Numerical |
| *Other lifestyles* | Smoking | 0: never smoked, < 100 cigarettes  1: ≥ 100 cigarettes | Categorical |
|  | Perceived stress level | 0: rarely, 1: sometimes, 2: often, 3: very often | Categorical |
|  | Average sleep time per day | Range: 1–13 | Numerical |

Supplementary Table 1. Refined value forms for machine learning

Abbreviations: **ML**: Machine learning.

Supplementary Table 2. Description of the machine learning model

| **Machine-Learning Models** | **Descriptions** |
| --- | --- |
| Logistic regression | Logistic regression is used exclusively for classifications, which are performed by transforming the output between 0 and 1 using a transformation called the logistic function. The coefficients of the logistic regression (LR) equations are calculated using training data. |
| K-nearest neighbor classifier | The KNN classifier adapts lazy learning that classifies datasets based on their similarities with neighbors. ‘K’ stands for the neighbors near the test point and picks the popular class among them. ‘K’ is usually odd to avoid anomalies. |
| Naive Bayesian | Naive Bayes (NB) is one of the most efficient and effective inductive learning algorithms for machine learning and data mining. Naive Bayesian is a classification technique based on Bayes' theorem, assuming independence among predictors. It is a probabilistic classifier that calculates the probability of a given data point belonging to each class and then selects the class with the highest probability. |
| Decision tree classifier | The decision tree is a popular machine-learning algorithm for classification and regression tasks. It is a tree-like structure where each internal node represents a "decision" based on the value of a feature, each branch represents the outcome of that decision, and each leaf node represents the final decision or outcome. |
| Random forest classifier | The random forest (RF) classifier adapts ensemble learning algorithms. In this machine-learning classifier type, Bootstrap aggregating or Bagging is used in the training algorithm. |
| Support vector machine (SVM) classifier | The support vector machine (SVM) classifier utilizes the extremes of the hyperplanes dataset. Given a set of labeled training data, SVM aims to find the hyperplane that best separates the data into different classes. This hyperplane is a line in two dimensions; in higher dimensions, it is a hyperplane. |
| XGBoost | The XGBoost, which stands for extreme gradient boosting, is a scalable, distributed gradient-boosted decision tree (GBDT) machine-learning library. It provides parallel tree boosting and is the leading machine-learning library for regression, classification, and ranking problems. |
| LightGBM | The LightGBM (LGB) is a fast gradient-boosting framework designed for large datasets. It constructs models by boosting trees, excelling in both speed and accuracy. It handles categorical data well and requires thoughtful tuning for optimal performance. |
| CatBoost | The CatBoost is a gradient-boosting classifier on [decision trees](https://builtin.com/machine-learning/decision-tree), meaning it can be used for classification, [regression](https://builtin.com/learn/courses/regression-data-mining-text-mining-forecasting-using-r), and ranking tasks. CatBoost combines ordered boosting, random permutations, and gradient-based optimization to achieve high performance on large, complex datasets with categorical features. |

Abbreviations: **CatBoost:** Category boosting; **LightGBM:** Light Gradient-Boosting Machine; **XGBoost:** Extreme Gradient Boosting

Supplementary Figure 1. The permutation importance feature.

Supplementary Figure 1.1. Logistic regression.


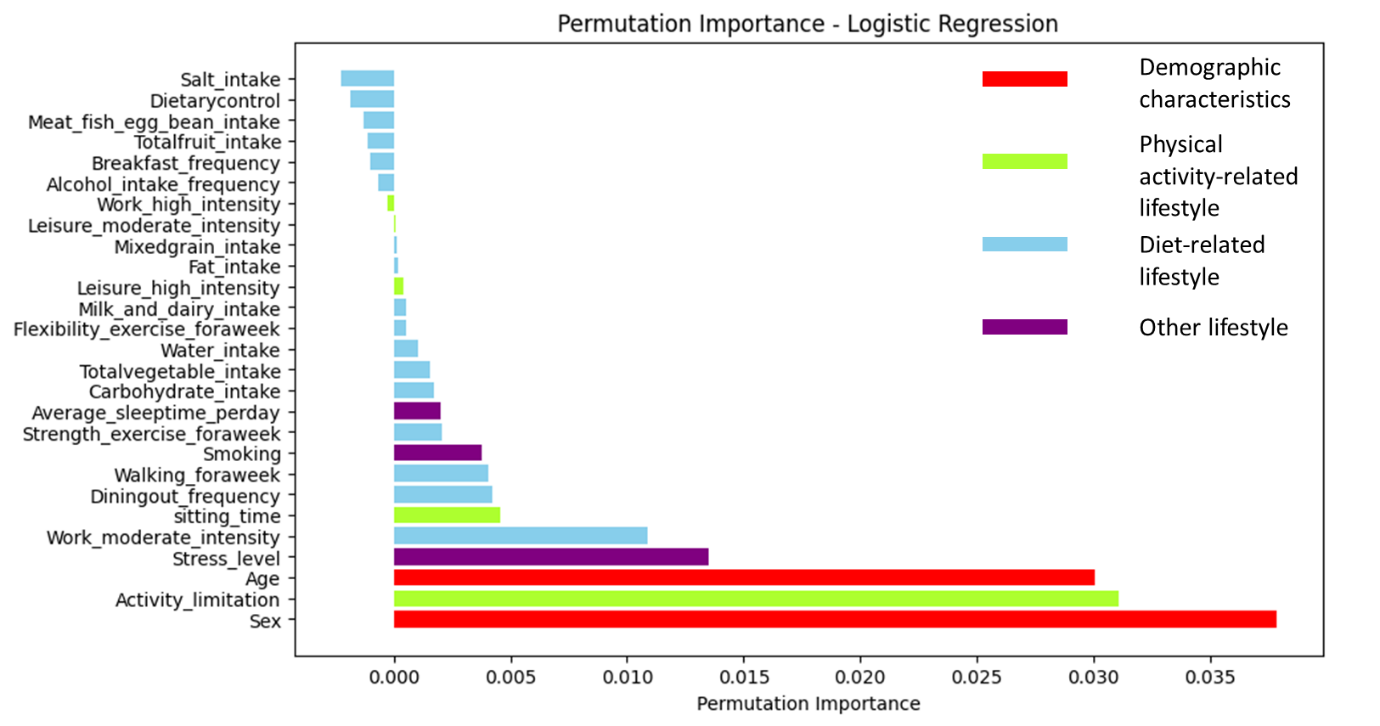


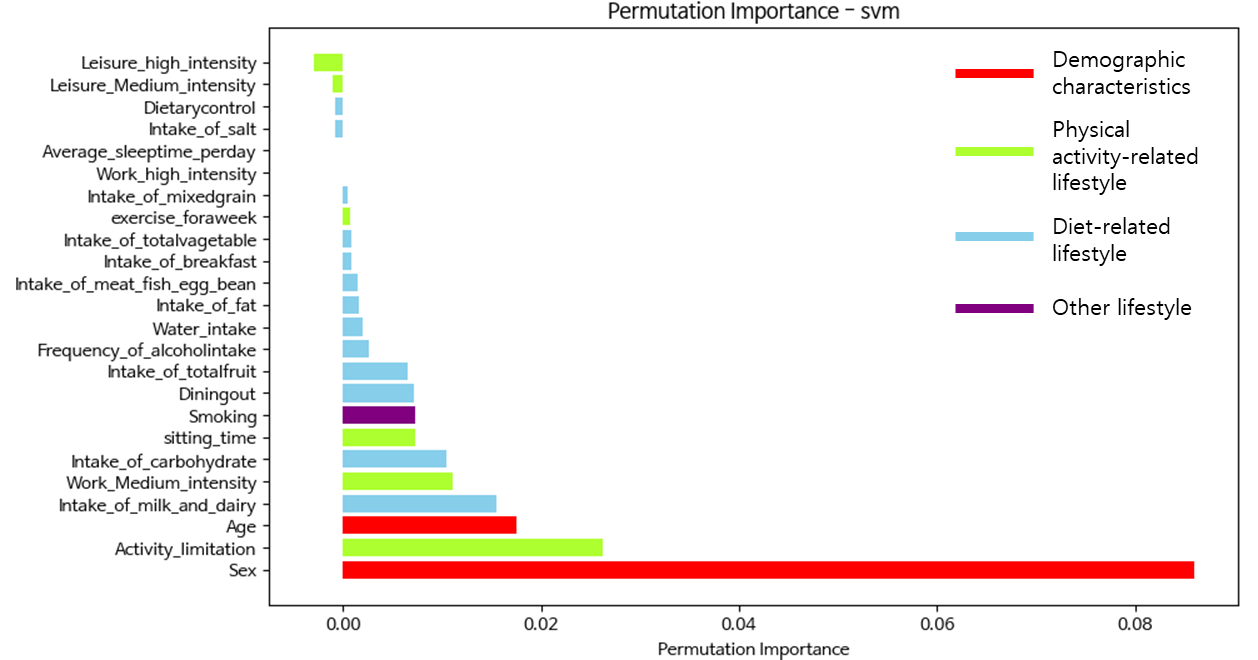


Supplementary Figure 1.2. Support vector machine (SVM).


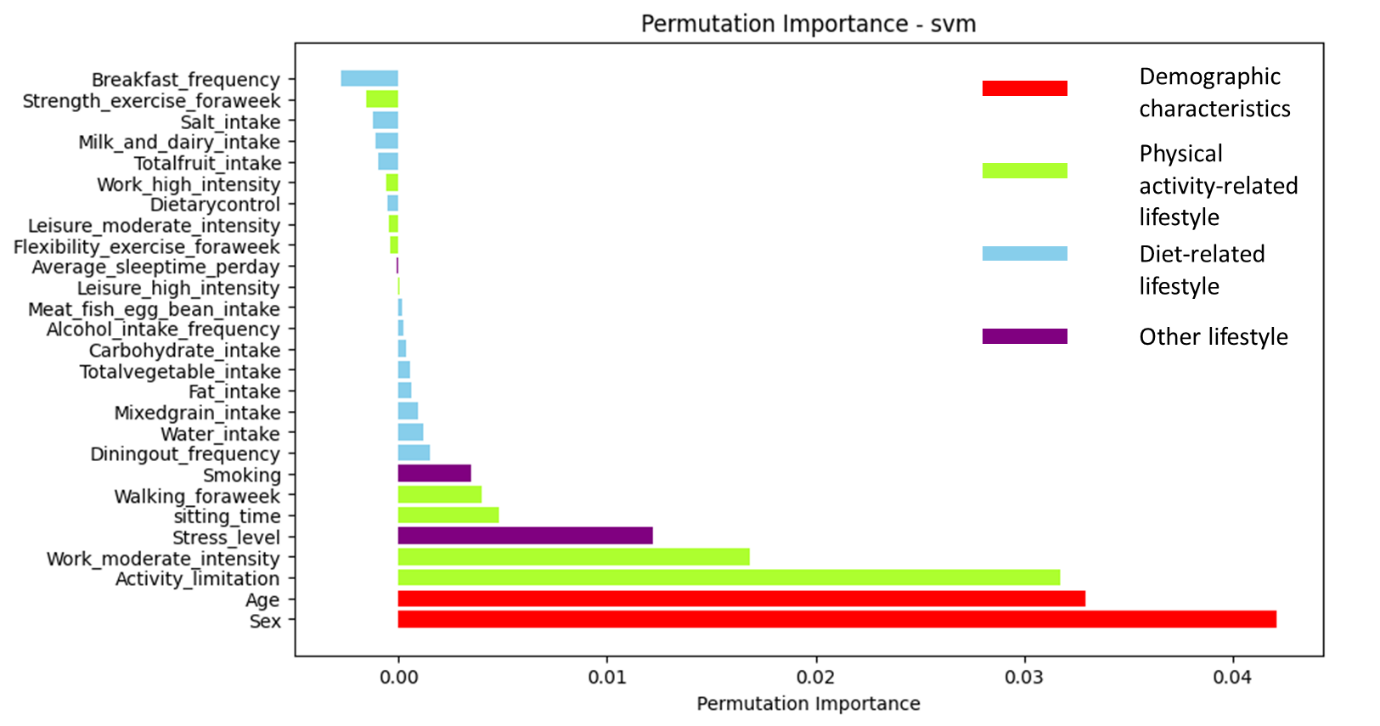


Supplementary Figure 1.3. Random forest.


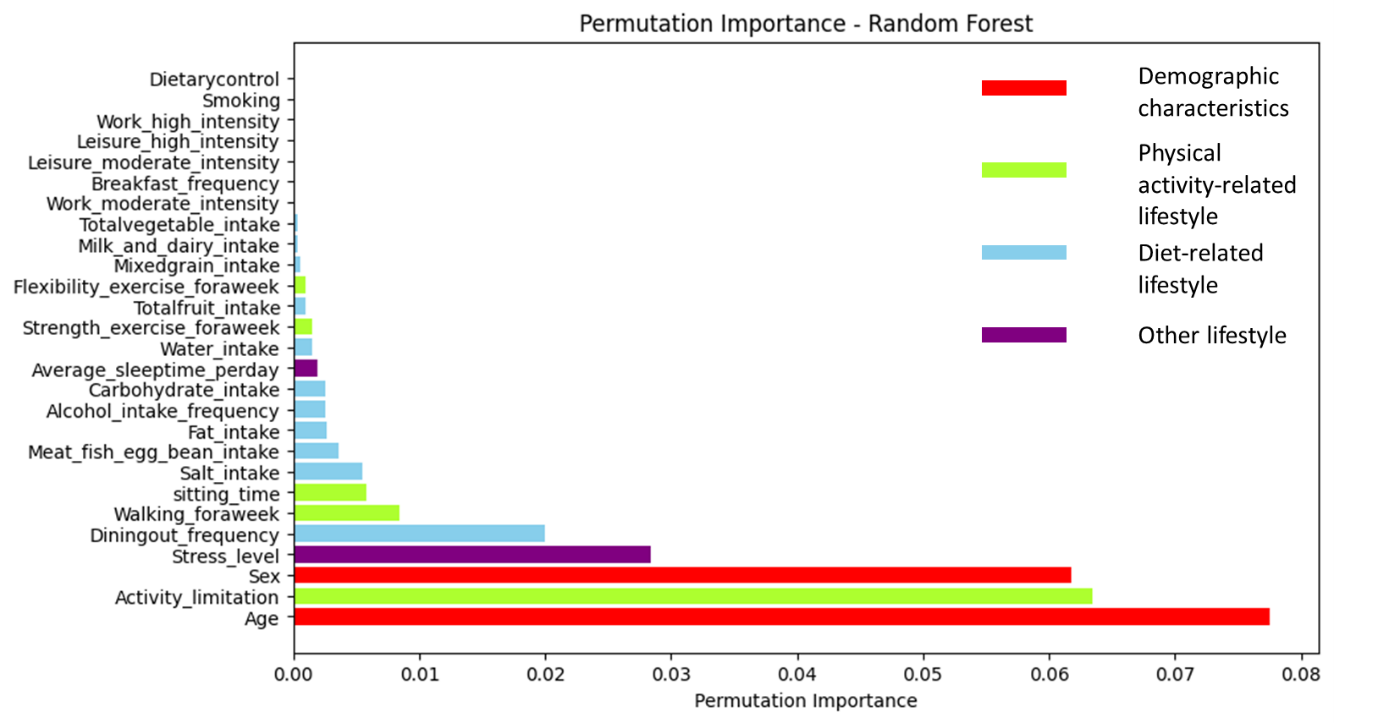


Supplementary Figure 1.4. Naive Bayesian.


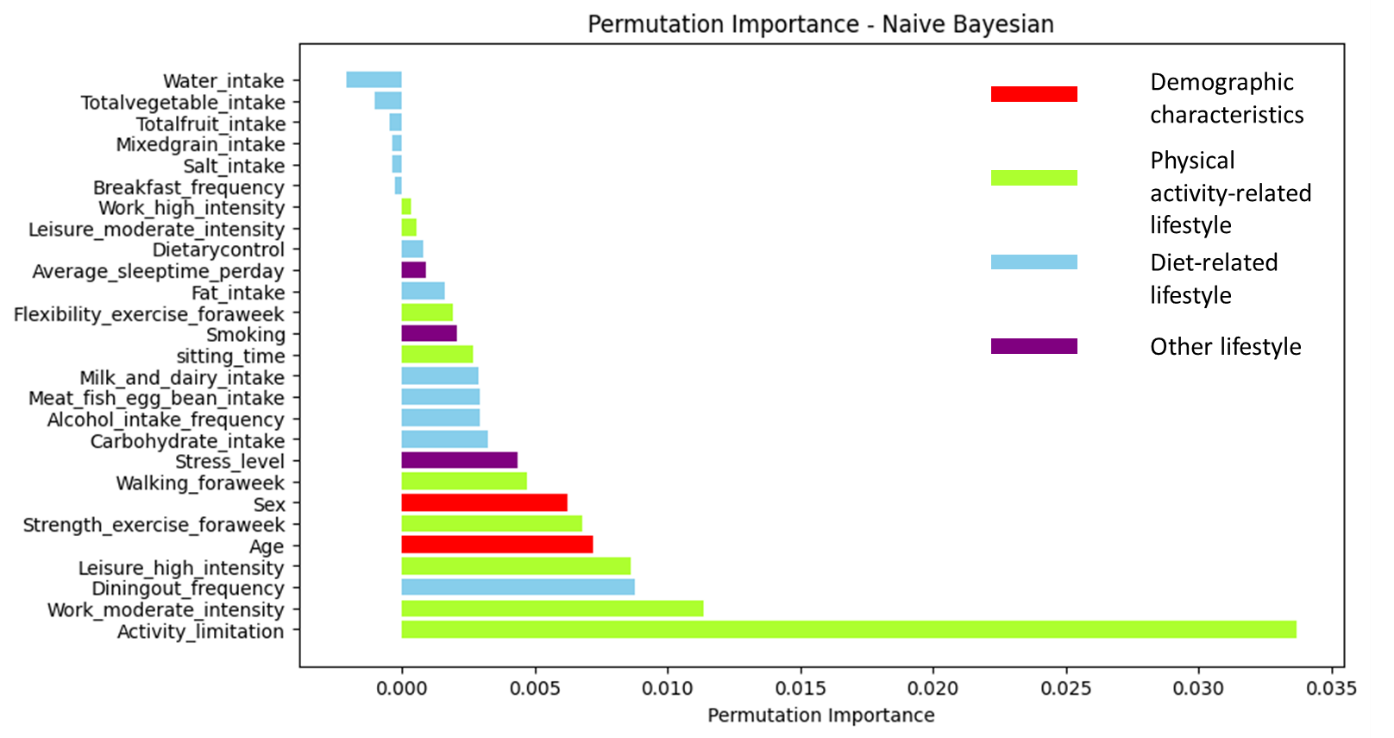


Supplementary Figure 1.5 CatBoost.


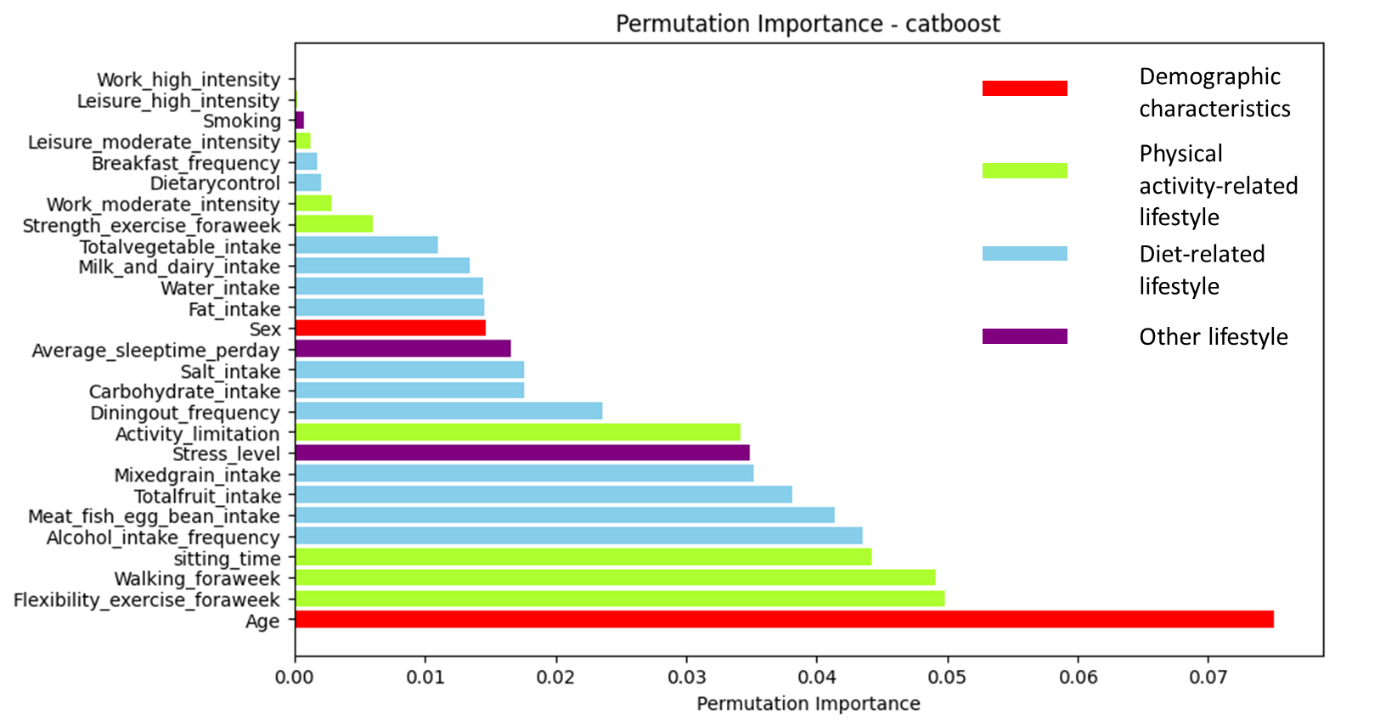


Supplementary Figure 1.6. XGBoost.


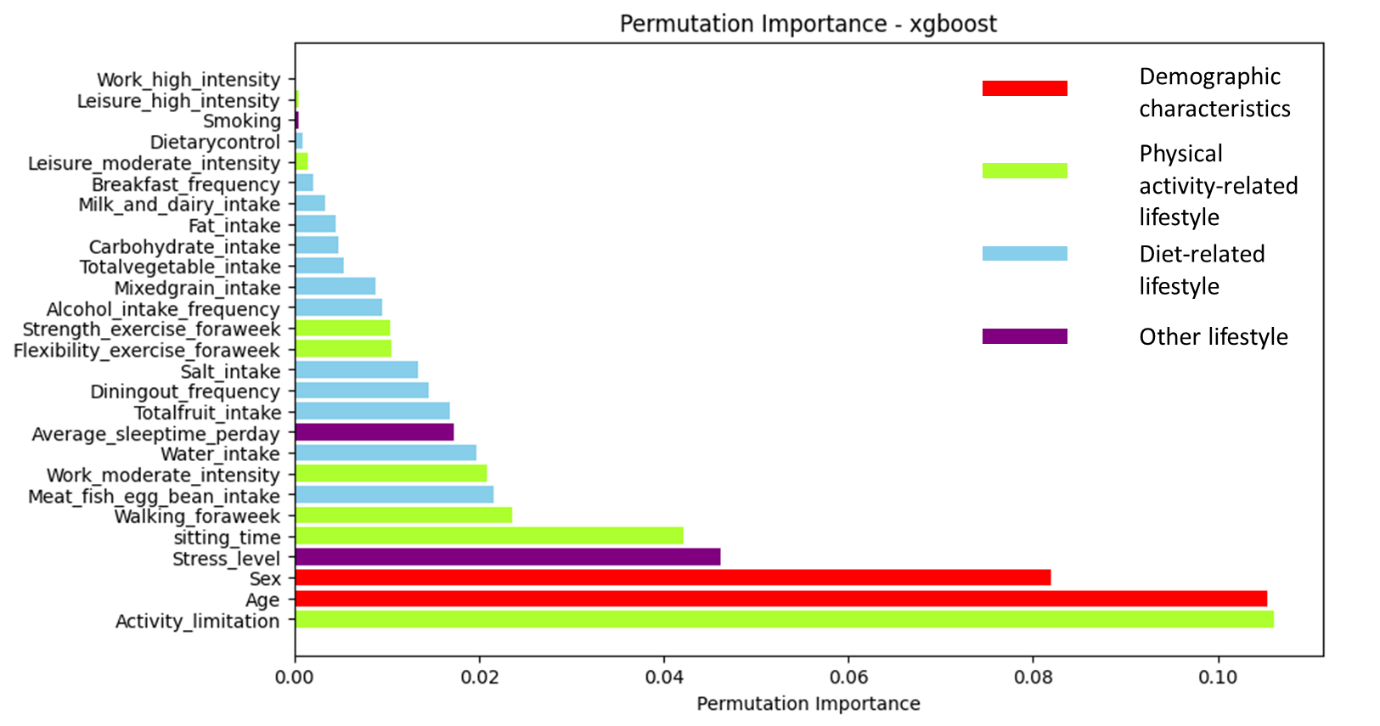


Supplementary Figure 1.7. LightGBM.
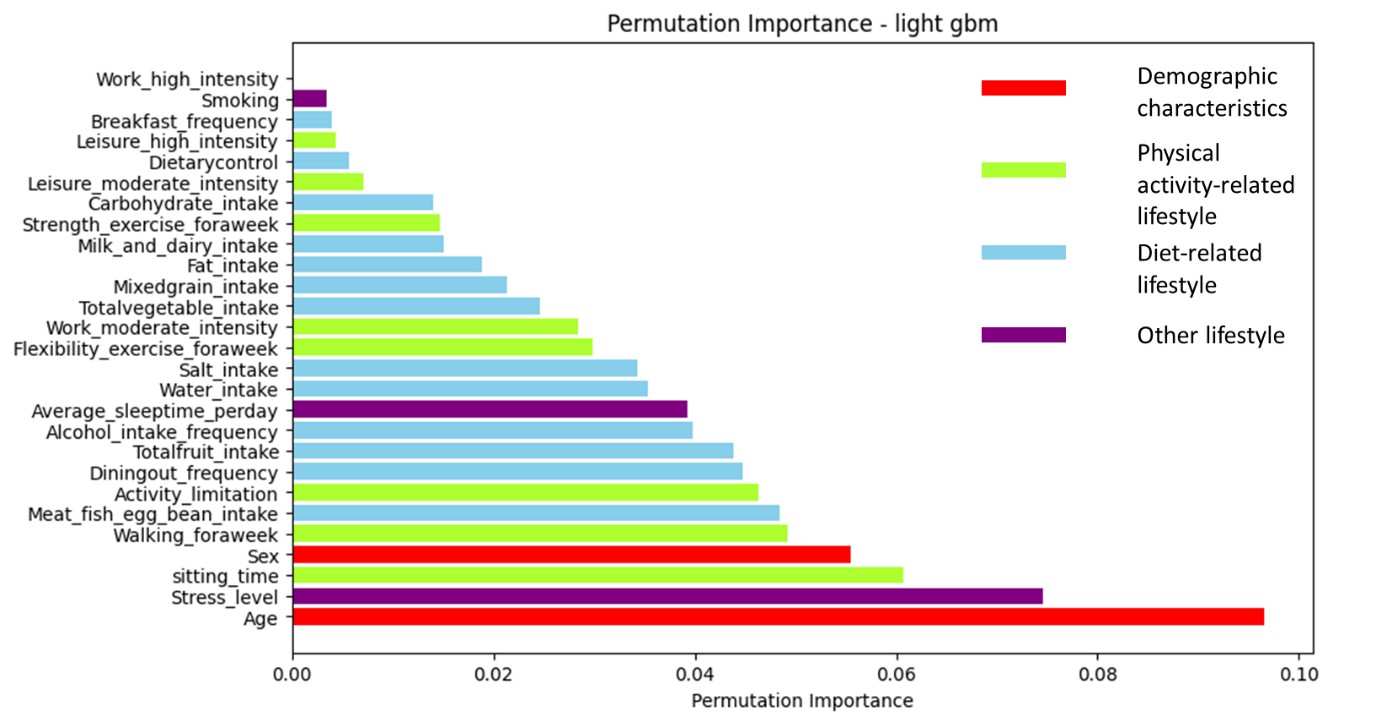


Supplementary Figure 1.8. K-nearest neighbor (KNN).


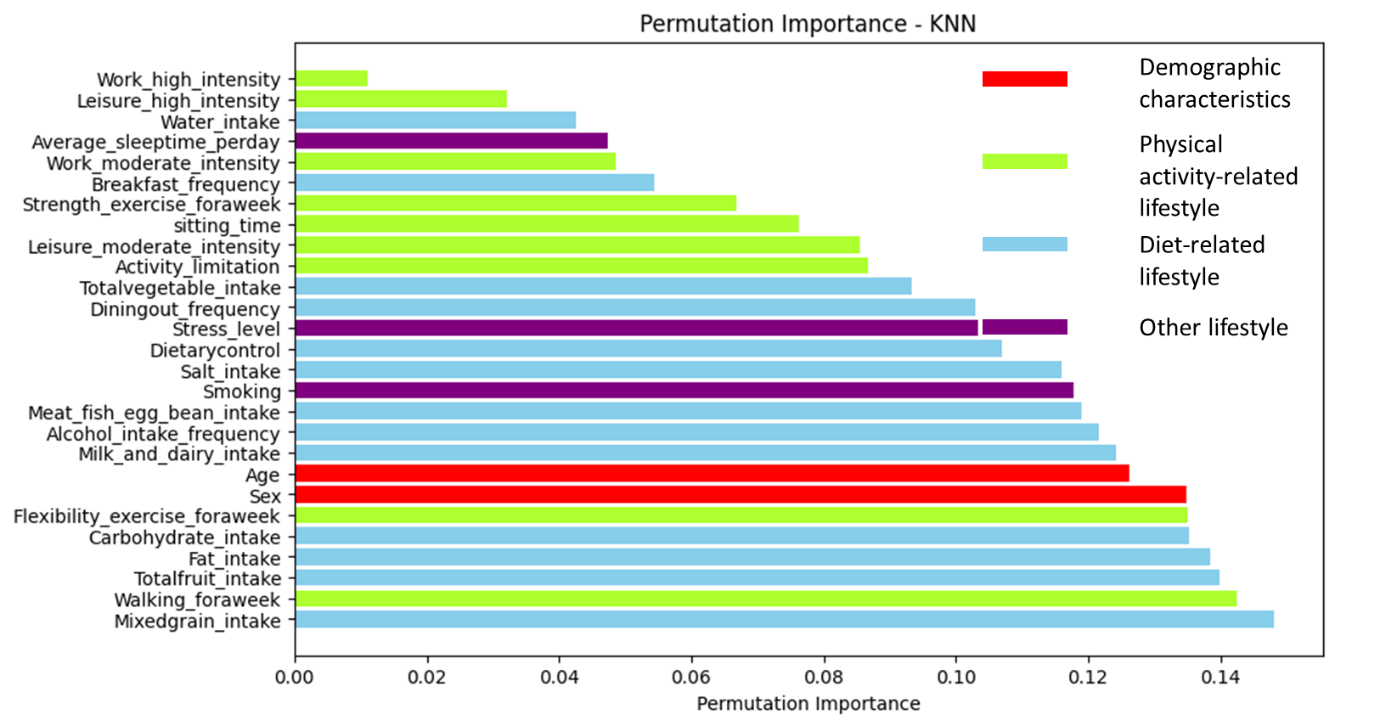


Supplementary Figure 1.9. Decision tree.
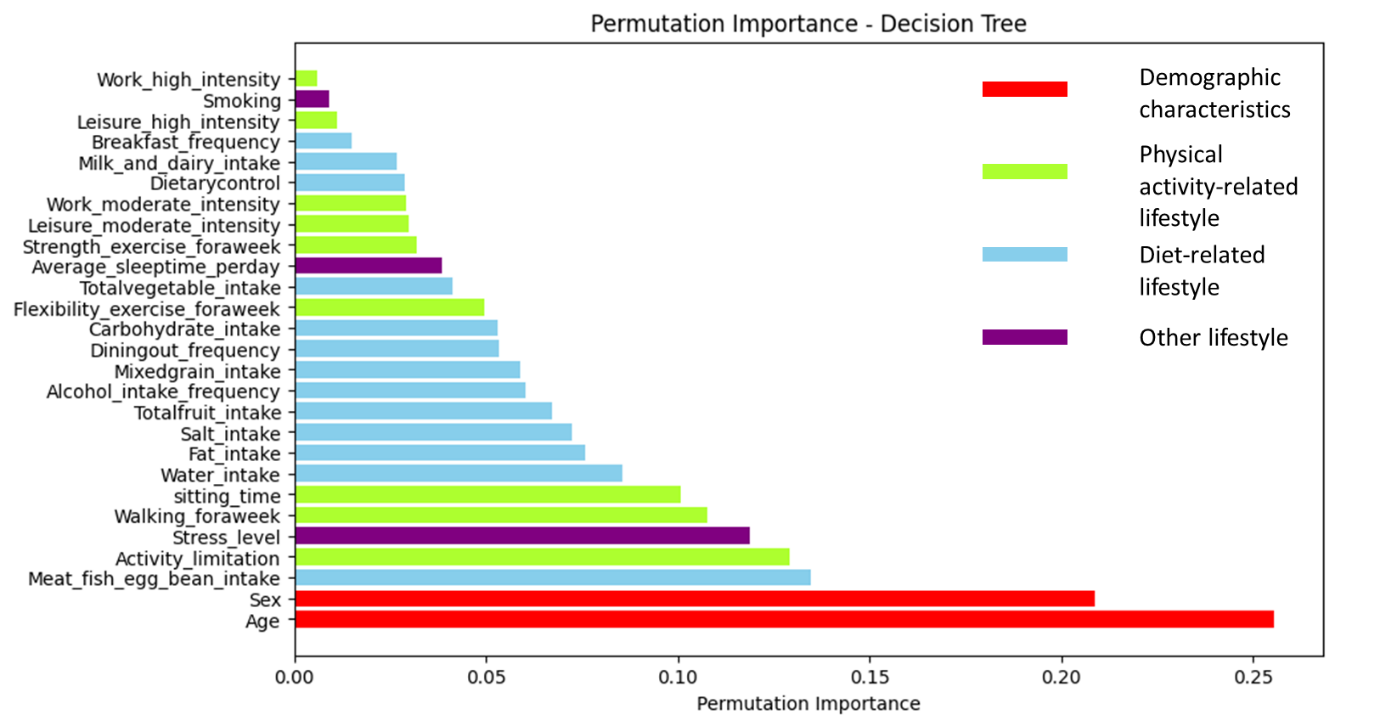


Supplementary Figure 2. SHAP value plot of Logistic Regression.


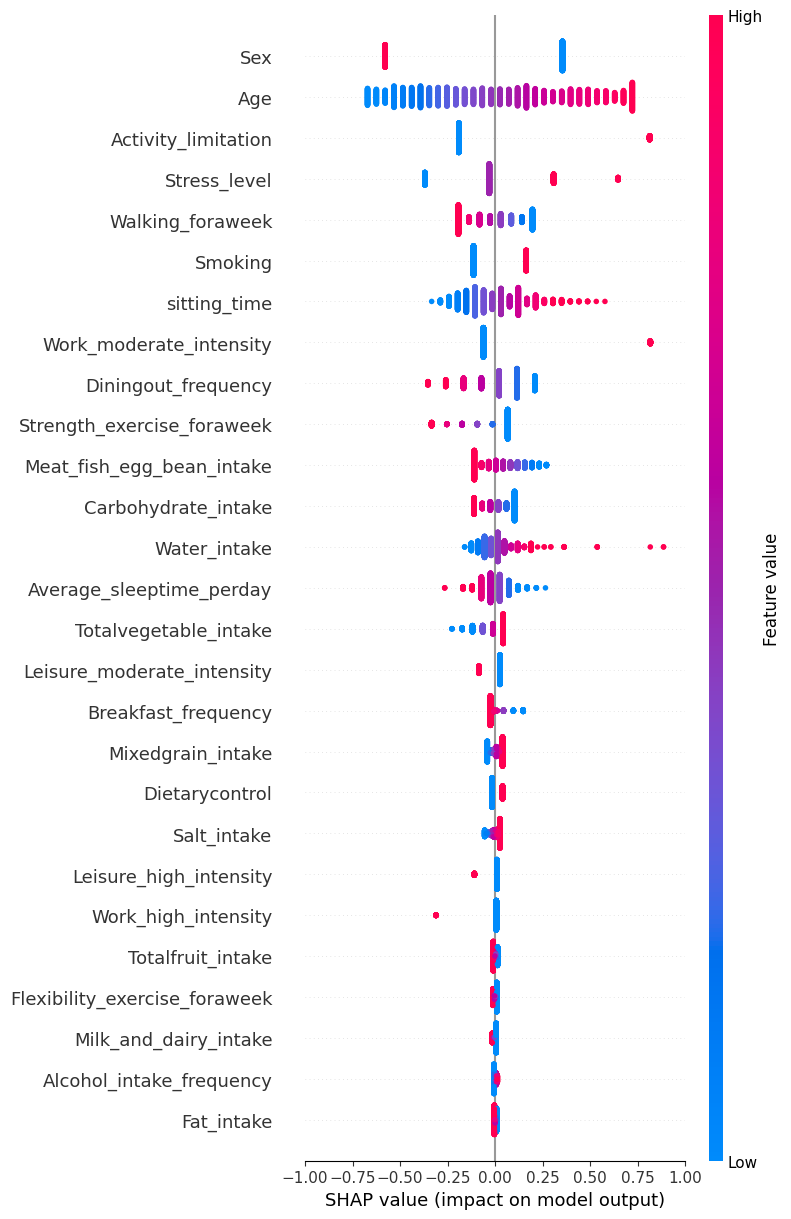

Supplement: Supplementary file 1 — Table S1: Refined value forms for machine learning. Table S2: Description of the machine learning model. Figure S1: The permutation importance feature. Figure S1:1 Logistic regression. Figure S1:2 Support vector machine (SVM). Figure S1:3 Random forest. Figure S1:4 Naive Bayesian. Figure S1:5 CatBoost. Figure S1:6 XGBoost. Figure S1:7 LightGBM. Figure S1:8 K‐nearest neighbor (KNN). Figure S1:9 Decision tree. Figure S2: SHAP value plot of logistic regression. [file NHS-27-e70248-s001.docx]
